# Editorial: Clinical application of artificial intelligence in emergency and critical care medicine, volume V

**DOI:** 10.3389/fmed.2025.1593416

**Published:** 2025-04-11

**Authors:** Yanni Wang, Rahul Kashyap, Ping Zhang, Qinghe Meng, Zhongheng Zhang

**Affiliations:** ^1^Department of Emergency Medicine, The Second Affiliated Hospital of Anhui Medical University, Hefei, Anhui, China; ^2^Department of Emergency Medicine, Sir Run Run Shaw Hospital, Zhejiang University School of Medicine, Hangzhou, China; ^3^Division of Pulmonary and Critical Care Medicine, Mayo Clinic, Rochester, MN, United States; ^4^Department of Research, WellSpan Health, York, PA, United States; ^5^Department of Computer Science and Engineering, The Ohio State University, Columbus, OH, United States; ^6^Department of Biomedical Informatics, The Ohio State University, Columbus, OH, United States; ^7^Department of Surgery, State University of New York Upstate Medical University, Syracuse, NY, United States; ^8^Sepsis Interdisciplinary Research Center, State University of New York Upstate Medical University, Syracuse, NY, United States; ^9^School of Medicine, Shaoxing University, Shaoxing, Zhejiang, China; ^10^Key Laboratory of Precision Medicine in Diagnosis and Monitoring Research of Zhejiang Province, Sir Run Run Shaw Hospital, Zhejiang University School of Medicine, Hangzhou, China; ^11^Longquan Industrial Innovation Research Institute, Lishui, China

**Keywords:** artificial intelligence, prediction, machine learning, critical care, treatment decision-making

This Research Topic explores the clinical applications of artificial intelligence (AI) in emergency and critical care medicine through 13 research articles. These studies systematically investigate how advanced data mining and AI techniques enhance risk assessment, diagnostic assistance, prognosis prediction, and treatment decision-making. Leveraging large-scale data resources and machine learning (ML) algorithms, these studies provide detailed analyses of complex conditions such as sepsis, acute pancreatitis, gastroparesis, acute kidney injury, disseminated intravascular coagulation (DIC), and heart failure with sepsis. From a data-driven perspective, this research offers a robust theoretical foundation and practical support for precision medicine.

AI is expected to significantly improve the prognosis of critically ill patients by assisting in disease identification, predicting disease progression, and supporting clinical decision-making (1). One key area of focus is the early prediction of sepsis, Yadgarov et al. conducted a systematic review of studies published from database inception to October 2023, searching Medline, PubMed, Google Scholar, and CENTRAL. Out of 3,953 studies, they analyzed 73 articles encompassing 457,932 sepsis patients and 256 models. Their findings demonstrated that ML models significantly outperformed traditional scoring systems in early sepsis prediction, with neural networks and decision tree models achieving the highest performance. Kim et al. developed a Transformer-based deep learning model for predicting ICU length of stay in sepsis patients, achieving a mean absolute error of only 2.05 days, demonstrating high accuracy and reliability. Beyond sepsis detection, Zhang et al. constructed a 28-day mortality prediction model specifically for sepsis patients with concurrent heart failure. Using the eICU-CRD database for model development and validating it externally on the MIMIC-IV database, they found that a logistic regression-based model achieved an AUC of 0.746 on the validation set, outperforming more complex algorithms such as XGBoost. The final model identified 10 key predictive features and employed the SHAP method to enhance interpretability, aiding clinicians in early identification of high-risk patients and optimizing resource allocation.

Traditional early warning systems primarily rely on vital signs and basic laboratory tests, whereas AI models integrating multi-source data allow for the incorporation of molecular and biological information into clinical decision-making. Hu et al. explored the combination of metagenomics, radiomics, and ML for sepsis diagnosis. By performing metagenomic sequencing on blood samples from sepsis patients and extracting radiomic features, they developed a fusion model that achieved an AUC close to 0.88 in its best-performing version. The integration of multimodal data helps overcome the limitations of single-indicator approaches, providing a novel strategy for the early and precise diagnosis of sepsis.

Furthermore, interpretable AI-based risk prediction models offer refined and individualized decision support in critical care medicine (2). Zhai et al. developed an interpretable XGBoost model for predicting in-hospital mortality risk in patients with severe pulmonary infections, achieving an AUC of 0.956. More importantly, the model incorporated SHAP and LIME methodologies to enhance interpretability. Such personalized risk assessment tools assist in mortality risk stratification and provide valuable clinical decision support. However, for AI models to truly assist in ICU decision-making, they must evolve from purely “predictive AI” to “actionable AI” capable of comparing the effects of different interventions. This transition necessitates the integration of causal inference to guide optimal treatment selection (3).

Beyond sepsis, AI technologies also hold significant clinical potential in other critical conditions. Tan et al. reviewed advancements in ML applications for predicting disease severity and complications in acute pancreatitis. Liu et al. utilized explainable ML to construct a postoperative gastroparesis risk prediction model, providing empirical evidence for early intervention. In another study, Wei et al. developed a nomogram for predicting early acute kidney injury (AKI) in patients with acute non-variceal upper gastrointestinal bleeding (NVUGIB) using data from the MIMIC-IV database, subsequently implementing it as a web-based clinical calculator.

AI also demonstrates immense potential in personalized treatment strategies. Traditional clinical guidelines often struggle to account for the unique physiological characteristics of individual patients, whereas AI-driven approaches offer the ability to support clinicians in devising more precise and tailored treatment plans (4). Nevertheless, while AI has demonstrated outstanding performance, it is crucial to recognize its limitations and reinforce validation and regulatory oversight in real-world applications to ensure tangible patient benefits (5).

Overall, *Clinical application of artificial intelligence in emergency and critical care medicine, Volume V* presents a comprehensive workflow from data acquisition and feature extraction to model construction and result interpretation. With advancements in multimodal data integration, causal inference modeling, and improvements in AI interpretability, AI is poised to become a powerful tool in sepsis management. We anticipate the deep integration of AI technologies into clinical medicine, fostering more precise and efficient workflows from early disease identification to treatment decision-making, ultimately contributing to lower mortality rates worldwide.
